# Glutathione peroxidase 4 inhibition induces ferroptosis and mTOR pathway suppression in thyroid cancer

**DOI:** 10.1038/s41598-022-23906-2

**Published:** 2022-11-12

**Authors:** Konjeti R. Sekhar, David N. Hanna, Sriram Cyr, Jordan J. Baechle, Sudhakiranmayi Kuravi, Ramesh Balusu, Kimryn Rathmell, Naira Baregamian

**Affiliations:** 1 Division of Surgical Oncology and Endocrine Surgery, Department of Surgery, Vanderbilt University Medical Center, 2220 Pierce Avenue, 597 Preston Research Building, Nashville, TN 37232 USA; 2 Vanderbilt University School of Medicine, Nashville, TN USA; 3 Division of Hematologic Malignancies and Cellular Therapeutics, Department of Internal Medicine, University of Kansas Medical Center, Kansas City, KS USA; 4 Division of Hematology and Oncology, Department of Medicine, Vanderbilt University Medical Center, Nashville, TN USA

**Keywords:** Cancer, Cell biology, Diseases, Endocrinology, Medical research, Molecular medicine, Oncology

## Abstract

Papillary thyroid carcinoma (PTC) demonstrates significantly reduced patient survival with metastatic progression. Tumor progression can be influenced by metabolism, including antioxidant glutathione (GSH). Glutathione peroxidase 4 (GPX4) is a selenoenzyme that uses GSH as a co-factor to regulate lipid peroxidation of cell membranes during increased oxidative stress. GPX4 suppression in tumor cells can induce ferroptosis. This study aims to examine ferroptosis as a potentially critical pathway in effective targeting of thyroid cancer (TC) cells. We treated human TC cells (K1, MDA-T68, MDA-T32, TPC1) with (1S,3R)-RSL3 (RSL3), a small-molecule inhibitor of GPX4 and examined the effects on ferroptosis, tumor cell survival and migration, spheroid formation, oxidative stress, DNA damage repair response, and mTOR signaling pathway in vitro. GPX4 inhibition activated ferroptosis, inducing TC cell death, rapid rise in reactive oxygen species and effectively arrested cell migration in vitro. Suppression of mTOR signaling pathway triggered autophagy. GPX4 genetic knockdown mirrored RSL3 effect on mTOR pathway suppression. RSL3 subdued DNA damage repair response by suppressing phosphorylation of nucleophosmin 1 (NPM1). Thus, observed potent induction of ferroptosis, GPX4-dependent novel suppression of mTOR pathway and DNA damage repair response in preclinical in vitro model of TC supports GPX4 targeting for therapeutic benefit in advanced therapy-resistant thyroid cancers.

## Introduction

Thyroid cancer is currently the predominant form of endocrine cancer in the world, accounting for greater than 90% of all endocrine malignancies, with an increasing incidence in the United States^1,2^. The most common type of thyroid cancer is papillary thyroid carcinoma (PTC)^1,3^. Treatment of PTC has traditionally involved surgery and radioactive iodine ablation therapy. These treatment modalities are generally effective, with low recurrence rates between 1 and 3%^4^. However, a small subset of patients presents with highly aggressive or radioactive iodine-resistant forms of thyroid cancers with increasing recurrence rates of nearly 15% within 10 years^5^. Tumor progression into soft tissues, lymph nodes or distant metastatic sites have been shown to dramatically reduce 5-year overall survival in patients with PTC, highlighting the need for a novel therapeutic approach for highly aggressive forms of PTC^6^.

In recent years, ferroptosis has emerged as a promising target for countering highly aggressive malignancies, and is an iron-dependent form of regulated cell death associated with accumulation of reactive oxygen species (ROS) and increased lipid peroxidation^7^. Transferrin receptor 1 (TfR1), a well-known regulator of cellular iron that internalizes the transferrin-bound iron, plays a significant role in ferroptosis, and ROS induction has been shown to enhance TfR1 protein levels and serves as a marker of ferroptosis^8^. While many of the key regulators of this pathway remain largely unknown, recent studies have identified glutathione peroxidases, particularly glutathione peroxidase 4 (GPX4), as antioxidant enzymes that can prevent lipid peroxidation using reduced glutathione (GSH) as a co-factor^9,10^. Previous studies have shown that a small molecule called *Ras* Lethal 3 ((1S,3R)-RSL3,(RSL3)) can pharmacologically inhibit GPX4 in skin and soft tissue cancer models in vitro and leads to induction of ferroptosis^10,11^. To date, no study has pharmacologically targeted the ferroptosis pathway in PTC by RSL3-mediated suppression of GPX4 activity.

GPX4 serves multiple roles in its inhibition of iron-dependent ferroptosis regulated cell death^12–14^. The capacity of GPX4 to influence cell viability through several pathways highlights its potential as a promising therapeutic target for malignancies. The mechanism by which it is regulated and potentially inhibited promises novel avenues for treatment of aggressive types of malignancies^15–19^. Studies have identified interactions between the mTOR signaling pathway and GPX4 expression, demonstrating that use of the classical ferroptosis activator, RSL3, can additionally block mTOR activation^9^. The mTOR pathway plays a key role in cancer activity and has also been shown to prevent iron-mediated cell death by regulating ROS production^20^. In PTC tumors, the BRAF^V600^^E^ mutation has been shown to be positively correlated with mTOR pathway activation and upregulation^21^. Therefore, examining mTOR pathway suppression by targeting GPX4 can provide further mechanistic insight into mTOR pathway signaling as a potential therapeutic target for advanced thyroid cancers.

In this study, we demonstrate that GPX4 is overexpressed in thyroid cancer and is associated with reduced overall survival due to its ability to prevent ferroptosis. Pharmacologic and genetic suppression of GPX4 induces ferroptosis, DNA damage, and decreased cell viability in vitro via a novel mechanism of mTOR signaling pathway inhibition.

## Results

### GPX4 overexpression negatively impacts overall survival in patients with papillary thyroid carcinoma

**Figure 1:**
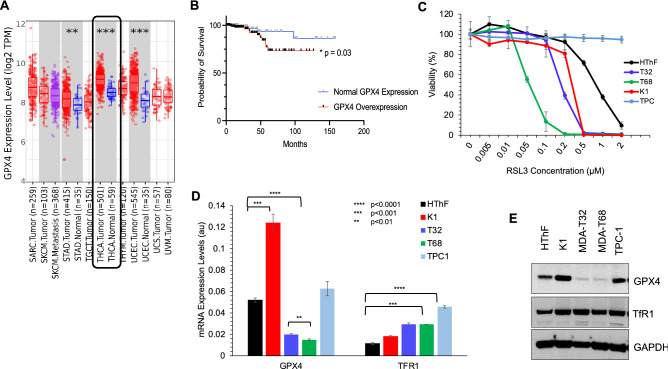
GPX4 and TfR1 expression levels in papillary thyroid carcinoma. (**A**) TIMER2.0 data analysis from TCGA database of Papillary Thyroid Carcinomas (N = 501) showing estimated *GPX4* mRNA expression levels in thyroid cancer (black box) compared to the normal thyroid tissue (N = 59) (log_2_ fold change 9.7 vs 8.2, *p* < 0.001). (**B**). GPX4 overexpression is associated with worse 5-year OS (73% vs 97%; HR 2.95; 95% CI 2.1–7.9; *p* = 0.03). (**C**) Viability assay of thyroid cancer cell lines K1, MDA-T32, MDA-T68, TPC-1 and HThF control cells after treatment with RSL3 for 48 h. (**D**) RT-qPCR for *GPX4* and transferrin receptor 1 (*TfR1*) mRNA levels, normalized to β-actin, in K1, MDA-T32, MDA-T68, and TPC-1 cancer cell lines, as well as human thyroid fibroblasts (HThF) (*p < 0.05, **p < 0.01, ***p < 0.001, ****p < 0.0001, ns—*p* > 0.05). **E** Protein levels for *GPX4* and *TfR1* measured by Western blot analysis.

We assessed the GPX4 enzyme expression levels in human PTC. We queried the TCGA database of human PTC tumor specimens (N = 501) using TIMER 2.0 computational platform. *GPX4* was overexpressed in PTC tissue samples relative to 59 benign thyroid control tissues (log^2^ fold change 9.7 vs. 8.2, *p* < 0.001) (Fig. 1A). Patients with normalized *GPX4* expression > 0.0 were considered to have *GPX4* overexpression in 226 (45.1%) patients. mRNA expression data and survival data were available for 96% of patients (n = 488) in the TCGA database with PTC. Patients with *GPX4* overexpression had a reduced 5-year OS of 73% compared to 97% in patients with normal *GPX4* expression (HR 2.95; 95% CI 2.1–7.9; *p* = 0.03) (Fig. 1B). These findings indicate that enhanced GPX4 expression is prevalent in thyroid cancer and is associated with reduced 5-yr overall survival.

### RSL3 suppresses viability in thyroid cancer cell lines

To determine whether GPX4 inhibition affects tumor cell viability, we treated PTC cells with various concentrations of RSL3 and performed a cellular viability assay. Additionally, we sought to demonstrate specificity of RSL3 for targeting *BRAF/RAS* addicted cancer cells by selecting TPC-1, a PTC cell line, that lacks *RAS* or *BRAF* mutations. The lethal concentration (LC_50_) for HThF cells was ~ 1 μM, while the LC_50_ for the PTC cell lines was substantially less (0.05–0.5 μM). The LC_50_ values for MDA-T68 and MDA-T32 cells were ~ 0.05 μM and 0.2 μM, respectively; LC_50_ for K1 cells was ~ 0.4 μM, whereas RSL3 treatment did not significantly affect TPC-1 cell viability, suggesting RSL3 selectively inhibits *RAS/BRAF*-mutant cell lines, such as K1, MDA-T32, and MDA-T68 (Fig. 1C).

### Variable GPX4 and TfR1 expression levels in papillary thyroid cancer cell lines

To examine the relative expression of GPX4 in thyroid cancer cell lines, we performed RT-qPCR on K1, MDA-T32, MDA-T68, and TPC-1 cells, and control human thyroid fibroblasts (HThF). Among the 4 cancer cell lines, K1 cells exhibited higher *GPX4 *gene expression relative to MDA-T32, MDA-T68, and TPC-1 cancer cells and control HThF. We also observed increased gene expression levels of Transferrin receptor 1 (*TfR1)* in all four cancer cell lines relative to HThF (Fig. 1D). Next, we analyzed protein expression of GPX4 and TfR1 by Western blotting.

GPX4 and TfR1 proteins were overexpressed in some TC cell lines compared to HThF cells. K1 cells appeared to have much higher expression of these proteins compared to MDA-T32, MDA-T68 and TPC-1 cells (Fig. 1E, and Supplemental Fig. 1A). These in vitro findings corroborate the patient tumor tissue data (Fig. 1A) demonstrating GPX4 overexpression in 45% of patients with PTC. This analysis demonstrated that the expression levels of GPX4 and TfR1 can vary between thyroid cancer cell lines (Fig. 1C,D), suggesting underlying tumor heterogeneity at play, and that the GPX4 and TfR1 basal expressions may not serve as reliable markers in vitro.

### GPX4 inhibition with RSL3 induces ferroptosis, lipid peroxidation and reactive oxygen species (ROS) in thyroid cancer cells in vitro

**Figure 2:**
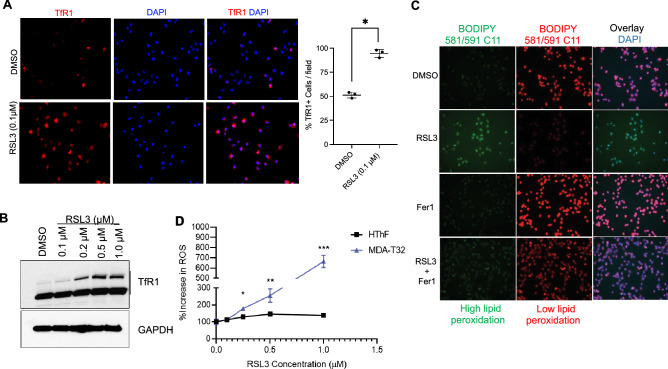
Pharmacologic inhibition of GPX4 with RSL3 induces ferroptosis activation, intracellular lipid peroxidation, and increased reactive oxygen species (ROS) in PTC in vitro. (**A**) Immunofluorescent staining for TfR1 in K1 cells treated with DMSO control or RSL3 (0.1 μM) for 6 h showed increased TfR1 expression relative to control-cells (94.7% vs 57.3%; *p* = 0.003; *TfR1* = red, DAPI = blue, 20 × magnification). (**B**) RSL3 induced increased TfR1 protein expression after 4 h treatment in dose-dependent manner. (**C**) Staining of K1 cells with BODIPY 581/591 C11 lipid peroxidation dye, a ferroptosis marker, in RSL3 (3 μM; 4 h) and ferrostatin-1 (Fer1, 5 μM)-treated cells. (**D**) Induction of intracellular reactive oxygen species (ROS) by RSL3 treatment (24 h) in MDA-T32 cancer cells and control HThF cells (*p < 0.05, ** p < 0.01, *** p < 0.001).

We targeted the ferroptosis pathway in PTC cells in vitro using a pharmacologic inhibitor of GPX4, RSL3. First, we investigated whether TfR1 levels were altered with RSL3 in PTC cells, as TfR1 has been shown to be a better marker of ferroptosis^22^. K1 cells were first treated with RSL3 for 6 h. Immunofluorescent (IF) staining of K1 cells was performed and revealed a significant increase in TfR1 protein expression compared to DMSO-treated control cells (RSL3 94.7% vs. DMSO 54.3%, *p* = 0.003) (Fig. 2A). K1 cells treated with increasing concentrations of RSL3 for 4 h demonstrated concentration-dependent increase in TfR1 protein levels (Fig. 2B). Similar observations were made in MDA-T32 and MDA-T68 PTC cell lines (Shown in Supplemental Fig. 2A and 2B).

Next, we demonstrated ferroptosis-induced accumulation of cellular lipid hydroperoxides following RSL3 treatment. RSL3-treated K1 cells were stained with lipid peroxidation sensitive dye BODIPY 581/591 C11 (Fig. 2C). With increased accumulation of lipid peroxides, the dye shifts its fluorescence from red (low peroxide levels) to green (high peroxide levels). RSL3-treated K1 cells had visibly increased lipid peroxidation relative to DMSO control. Ferrostatin 1 (Fer1), a known ferroptosis inhibitor, was used for co-treatment experiment and abrogated RSL3-mediated lipid peroxidation in PTC cells. Next, we measured RLS3-induced reactive oxygen species (ROS) levels in PTC cells. MDA-T32 cells exhibited RSL3-induced, disproportionately robust rise in intracellular ROS levels in concentration-dependent manner when compared to control HThF cells. (RSL3, 0.25 μM; 178.5% vs 129.3%; *p* = 0.03; RSL3, 0.5 μM; 255.2% vs 146.1%; *p* = 0.009; RSL3, 1 μM; 664.9% vs 138.2%, *p* < 0.001) (Fig. 2D). RSL3 treatment induced increased expression of ferroptosis marker TfR1 in several thyroid cancer cell lines compared to control (Figs. 2B, 5B, Supplemental Fig. 2A and 2B) despite varied basal levels (Fig. 1E), and significant rise in intracellular ROS and lipid peroxidation in thyroid cancer cells in vitro. Thus, we have demonstrated that RSL3 induces ferroptosis in thyroid cancer cells in vitro.

### RSL3 suppresses spheroid formation and viability, and halts migration of PTC cells in vitro

**Figure 3:**
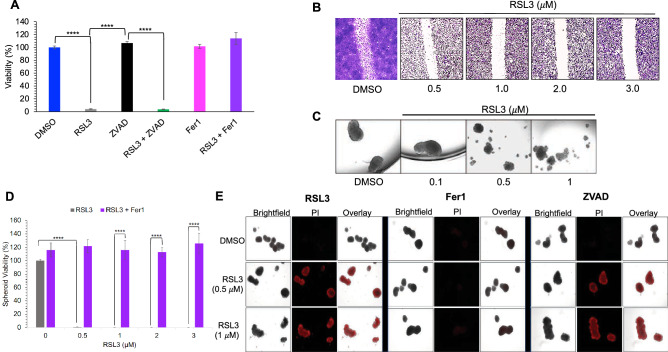
RSL3 suppresses K1 cell viability, K1 tumor cell spheroid formation and viability, and arrests thyroid tumor cell migration in vitro. (**A**) Viability of K1 cells treated with RSL3 (2 μM), Fer1, ZVAD, and combination of RSL3 with Fer1 or ZVAD. (**B**) Representative microscopy image showing arrested K1 cell migration after RSL3 treatment (× 10 magnification). (**C**) Representative microscopy image showing impaired spheroid formation of K1 cells after RSL3 treatment (× 20 magnification). (**D**) Viability of spheroids treated with RSL3 and Fer1 for 48 h were assessed (*p < 0.05, ** p < 0.01, *** p < 0.001, **** p < 0.0001, ns—*p* > 0.05). (**E**) Representative microscopic images showing propidium iodide (PI) staining of K1 spheroids treated with RSL3, Fer1 and ZVAD for 48 h.

We postulated that the RSL3-mediated suppression of thyroid tumor cell viability is a ferroptosis pathway-dependent process, therefore, we treated K1 cells with a lethal dose of RSL3 alone and in the presence of either a ferroptosis inhibitor Fer1, or a pan caspase inhibitor, Z-VAD-FMK (ZVAD) and measured cell viability. Fer1 treatment rescued PTC cells from RSL3-induced cell death, whereas ZVAD failed to exert similar effect (Fig. 3A), hence supporting our hypothesis that RSL3-induced PTC cell death is indeed a ferroptosis pathway-dependent phenomenon.

Next, we evaluated the impact of RSL3 treatment on PTC tumor cell migration and ability to form 3-dimensional (3D) PTC spheroids in vitro*.* K1 cells were treated with RSL3 at various concentrations (0.1 to 3.0 μM) and scratch assay showed significant halt in treated PTC cell migration (Fig. 3B). The 3D PTC spheroid formation was negatively impacted by the RSL3 treatment (Fig. 3C). Furthermore, we demonstrated that RSL3 not only suppressed the spheroid formation, but also reduced the viability of PTC spheroids (Fig. 3D). Moreover, we showed that Fer1 co-treatment protected K1 spheroids from RSL3-induced cell death, as determined by viability assay. RSL3-treated K1 spheroids (48 h) were stained with propidium iodide (PI) to visualize dead cells within the spheroids. As shown in Fig. 3E, DMSO-treated tumor cells have lower uptake of PI when compared to RSL3-treated PTC spheroids. When co-treated with Fer1, the PI uptake was effectively suppressed in the RSL3-treated 3D PTC spheroids, further reinforcing our previous findings of cytoprotective effects and ferroptosis pathway abrogation by Fer1 in RSL3-treated PTC cells in 2D. In contrast, the ZVAD co-treatment failed to suppress the uptake of PI in RSL3-treated 3D PTC spheroids, mirroring tumor cell viability findings in 2D. Collectively, our findings demonstrate that the GPX4 inhibition by RSL3 in PTC cells induces cell death, ferroptosis, lipid peroxidation, increased intracellular ROS burden, and significantly impairs thyroid tumor cell migration, 3D PTC spheroid fotmation and results in reduced spheroid viability in vitro.

### RSL3 induces DNA damage, inhibits DNA repair, and promotes autophagy in PTC cells

**Figure 4:**
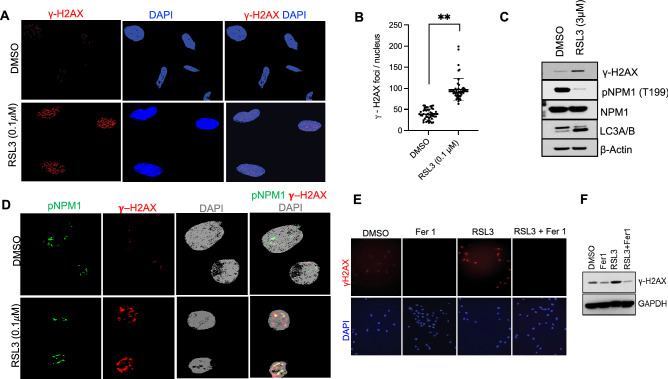
RSL3 induces double-stranded DNA breaks and inhibits DNA damage repair response in PTC in vitro. (**A**) Immunofluorescent staining for γ-H2AX nuclear foci expression in K1 cells treated with RSL3 or DMSO-control (γ-H2AX = red, DAPI = blue, × 20 magnification). (**B**) Quantification of γ-H2AX foci in 50 RSL3-treated K1 cells and 50 control-treated cells (RSL3, 97.3 foci/cell vs DMSO, 38.6 foci/cell; *p* < 0.0001). (**C**) Western blot analysis of γ-H2AX, pNPM1, NPM1, and LC3A/B in RSL3-treated or DMSO-treated K1 cells. (**D**) Dual immunofluorescent staining of K1 cells for pNPM1 (T199) and γ-H2AX co-localization using confocal microscopy (pNPM1 = green, γ-H2AX = red, DAPI = gray, × 63). (**E**) Immunofluorescent staining for γ-H2AX expression in RSL3-, Fer1- and DMSO-treated K1 cells (γ-H2AX = red, DAPI = blue, × 20 magnification). (**F**) Western blot analysis of γ-H2AX expression in RSL3-, Fer1- and DMSO-treated K1 cells.

We showed that the RSL3-mediated GPX4 inhibition induced multi-fold rise in intracellular ROS in PTC cells in vitro. Therefore, we anticipated increased ROS-mediated DNA damage in thyroid cancer cells. The IF staining and Western blot analyses for γ-H2AX, a marker for DNA double-strand damage, and phosphorylated nucleophosmin 1 (pNPM1) required for DNA damage repair were performed in RSL3-treated K1 cells. RSL3 (0.1 μM) treatment increased γ-H2AX levels and previously unreported reduced pNPM1 (T199) levels when compared to control treatment. The IF staining and confocal microscopy revealed significantly higher expression of nuclear γ-H2AX foci in RSL3-treated K1 cells (Fig. 4A). Quantification of fifty control and RSL3-treated PTC cells demonstrated a greater than two-fold increase in nuclear γ-H2AX foci with RSL3 treatment (RSL3, 97.3 vs DMSO, 38.6 foci/cell, *p* < 0.001) (Fig. 4B). Furthermore, the treated cells had increased co-localization of pNPM1 in the nucleus due to increased DNA damage. These findings were further corroborated by Western blot analysis, in which we observed increased γ-H2AX expression, decreased pNPM1 (T199) protein levels and increased expression of autophagosome LC3A/B proteins (Fig. 4C,D). Furthermore, Fer1 co-treatment restored γ-H2AX expression level in RSL-3 treated cells to that of the control cells (Fig. 4E,F). Together, these intriguing findings demonstrate that RSL3 can effectively induce DNA damage, impair DNA damage repair response, and trigger autophagy in thyroid cancer cells by inhibiting GPX4. Further studies aimed at elucidating the exact mechanisms underlying RSL3-induced DNA damage are needed.

### RSL3 inhibits mTOR signaling in thyroid cancer cells

**Figure 5:**
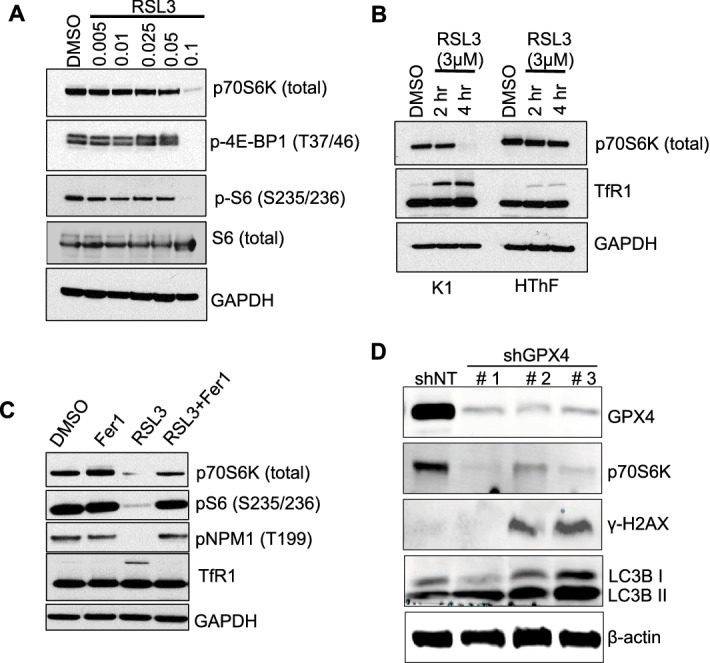
Activation of mTOR signaling pathway is inhibited by RSL3 and rescued with ferrostatin-1 in PTC in vitro. (**A**) Western blot showing expression of mTOR signaling pathway downstream proteins; p70S6K, p-4EBP1 and p-S6 in K1 cells treated with RSL3 or DMSO control for 6 h. (**B**) Western blot showing p70S6K and TfR1 expression in K1 cells or HThF cells treated with RSL3 (3 μM) or DMSO control for 2 and 4 h. (**C**) RSL3-mediated (3 μM) effect on mTOR pathway inhibition and induction of ferroptosis is abrogated by Ferrostatin-1 (5 μM) in K1 cells. (**D**) Western blot showing differences in GPX4, p70S6K, γ-H2AX, LC3BI & BII and beta-actin protein expression after GPX4 enzyme silencing with three different shRNA vectors and non-targeting control vector, shNT.

The mTOR signaling pathway has recently been implicated in counteracting ferroptosis by regulating ROS production^20^, therefore, it was important to examine the effects of RSL3 on mTOR signaling pathway and its downstream proteins and whether inactivation of mTOR pathway signaling will occur in thyroid cancer cells with GPX4 inhibition. K1 cells were again treated with various concentrations of RSL3 (0.005–3 μM), and Western blot analysis was performed. As anticipated, RSL3 treatment suppressed mTOR signaling pathway in PTC cells in vitro. In particular, we observed previously unreported, robust suppression of p70S6K, as well as suppression of p-4E-BP1 and p-S6 (Fig. 5 A, B, and Supplementary Fig. 3) downstream effectors of mTOR signaling pathway.

The mTOR directly phosphorylates 4E-BP1 and p70S6K which then phosphorylate S6 to initiate protein translation step. Interestingly, RSL3 did not suppress p70S6K protein levels in HThF cells to the extent observed in K1 cells (Fig. 5B). To demonstrate that suppression of p70S6K protein and pNPM1 by RSL3 is GPX4 inhibition-specific in PTC cells, we treated K1 cells with RSL3 (3 µM) in the presence of ferrostatin-1 (5 µM), an effective inhibitor of ferroptosis and analyzed by Western blotting. Fer1 alone did not alter p70S6K, pS6, pNPM1, or TfR1 expression; however, Fer1 exerted a profound rescue effect on the expression of these proteins after RSL3 treatment (Fig. 5C, Supplemental Figs. 1B and 4). Lastly, a genetic knockdown of *GPX4* with three different shRNA clones was performed, and p70S6K levels were measured by Western blotting. We observed robust inhibition of p70S6K levels in GPX4 knockdown cells compared to non-targeted cells (Fig. 5D). Similarly, we observed robust induction of γ-H2AX and LC3B I/B II levels with GPX4 genetic knockdown (Supplemental Fig. 5). These experiments reveal the multi-faceted impact of RSL3-mediated inhibition of GPX4 in thyroid cancer cells, by altering DNA damage repair and proliferation signaling pathways, and suppressing thyroid tumor cell growth.

## Discussion

A hallmark of cancer initiation and progression is evasion of regulated cell death, which contributes to therapy resistance. One such form, ferroptosis, the so called “death by iron”, is induced by the ROS and intracellular lipid peroxidation that is catalyzed by iron. This pathway has been studied in several in vitro models and is under investigation as a potential therapeutic target in several cancers, including breast, ovarian, liver, and prostate^15–17,23^. Previous studies have identified ferroptosis-related gene signatures as models to predict survival in patients^24,25^. To date, no study has targeted the ferroptosis pathway in thyroid cancer as a therapeutic option. Thyroid microenvironment has a high antioxidant metabolic profile at baseline due to inherently high intracellular ROS levels utilized for thyroid hormonogenesis. TIMER analysis of TCGA database revealed that GPX4 is overexpressed in many cancers. In this study, we show that nearly half of the human thyroid cancer specimens have increased GPX4 expression compared to control normal thyroid tissue and that the GPX4 overexpression is associated with worse overall survival. We have also demonstrated that GPX4 is highly expressed in some thyroid cancer cell lines. These findings have important clinical ramifications with the possibility of targeting ferroptosis pathway in GPX4-overexpressing thyroid cancers. Future studies are needed to reconcile the underlying differences between tumor tissue and basal PTC cell line GPX4 expression levels and in vitro responses to pharmacologic inhibition of GPX4 within an emulated 3D thyroid tumor microenvironment in vitro and in vivo.

The exact molecular mechanisms involved in the induction of ferroptosis and its downstream influence on cell survival are multifactorial. Our study is first to demonstrate novel molecular mechanisms involved in the sensitization of PTC cells to ferroptosis inducer- we show that RSL3, a potent direct GPX4 inhibitor, effectively reduces the viability of thyroid cancer lines with various mutational backgrounds compared to HThF control cell line in a dose-dependent manner. The PTC cell lines selected for the in vitro experiments represent some of the most common mutational signatures observed in thyroid cancer, particularly *BRAF*^*V600*^^*E*^ is found in more than 60% of PTCs and purports more aggressive tumor phenotype. The K1 cell line is a *BRAF*^*V600E*^*-PI3KCA* mutant, MDA-T32 is a *BRAF*^*V600E*^*-TERT* promoter mutant, and MDA-T68 is a *NRAS* and *TERT* promoter mutant, whereas TPC1 does not possess *RAS or BRAF* mutations.

The observed differential mRNA and protein expression levels of GPX4 and TfR1 in vitro may bear clinical relevance when considering factors such as tumor heterogeneity and the wide-ranging therapeutic response of thyroid tumors harboring diverse mutational signatures. The basal expression levels of GPX4 and TfR1 do not correlate with the sensitivity to RSL3 among PTC cell lines. Thus, there is a need to further characterize response differences between tumors when targeting ferroptosis pathway in thyroid cancer. Notably, PTC cell lines with relatively low basal GPX4 expression, such as MDA-T32 and MDA T-68 cells, had a lower LC_50_ compared to K1 cells with high GPX4 expression. This supports the fact that higher enzyme expression level may lead to higher LC_50_ values. The higher sensitivity of MDA-T32 and MDA-T68 cells to RSL3 treatment may be due to the fact that RSL3 was originally selected from the chemical screen that showed synthetic lethality against *RAS* mutation and active *TERT* expressing cells^26^. The MDA-T32 (*BRAF*-mutant) and MDA-T68 (*NRAS*-mutant) cell lines both express TERT mutation. On the other hand, *BRAF*-mutant K1 cells with *PIK3CA* E542K co-mutation exhibited surprising sensitization by RSL3. RSL3 has been shown to be inactive against other *PIK3CA* E542K mutated cell lines^27^. Wortmannin, a PI3K inhibitor, was unable to suppress RSL3 mediated cell death in *RAS* and *TERT*-expressing cells^26^. This is the first report to show that RSL3 is also synthetically lethal to the cells that harbor *PIK3CA* and *BRAF* mutation, such as K1 PTC cells.

We have shown that TPC-1 cell line did not respond to the RSL3 treatment when compared to *BRAF* or *RAS* mutant PTC cell lines, thus confirming that the effects observed in other PTC cell lines is in fact a GPX4-specific process. Results from the co-treatment with Fer1, a ferroptosis inhibitor, and ZVAD, an apoptosis inhibitor, indicate that RSL3 effects were reversed by Fer1 but not by anti-apoptotic ZVAD. In these studies, we have elucidated that RSL3 suppressed PTC spheroid formation, reduced spheroid viability, and these effects were reversed by Fer1 (Fig. 3D-F). The exact cellular mechanisms underlying the synthetic lethality of RSL3 on *BRAF/RAS* mutant PTC cells remain to be determined.

While RSL3 promotes ferroptosis via inhibition of GPX4, it also appears to enhance tumor cell DNA damage. Our study establishes that RSL3 contributes both to thyroid tumor cell DNA damage and concomitant inhibition of DNA damage repair response by limiting the availability of pNPM1 (T199). The recruitment of p-NPM1 and γ-H2AX at DNA damage sites is an essential step in the DNA damage recognition and DNA repair process. Immunofluorescent findings reveal that RSL3 alters the availability of two crucial proteins, which play important roles in DNA stability and repair. We observed co-localization of a phosphorylated NPM1 (T199), a protein that shuttles between the nucleolus and the nucleus^28,29^, and γ-H2AX, a histone protein that binds to DNA damage sites^30^ (Fig. 5D). With RSL3-induced reduction in pNPM1 levels, DNA damage sites (marked by γ-H2AX foci) were not repaired due to limited availability of pNPM1. Co-localization studies revealed that many of the DNA damage sites that are occupied by γ-H2AX did not co-localize with pNPM1 to efficiently repair all the damaged sites, which could in turn lead to tumor cell death. Our laboratory is the first to have observed RSL3-induced DNA damage and previously unreported suppression of DNA repair via limiting of pNPM1 levels. The exact mechanisms of pMPM1 suppression by RSL3 remains to be uncovered. RSL3 is known to interact with thioredoxin reductase 1 (Txnrd1) which also plays a role in DNA damage and oxidative stress^31^. However, lack of response to RSL3 treatment in non-mutant TPC-1 cells does not strongly support Txnrd1 role in DNA damage induced by RSL3 treatment. Additional studies are needed to definitively examine the role of Txnrd1 during ferroptosis in PTC cells. An interactive role between DNA damage and autophagy has been shown to play a critical role in the pathogenesis of colorectal cancer^32^. Several studies have shown that DNA damage can induce autophagy and vice versa. mTOR inhibitor, rapamycin, has been shown to increase radiation-induced DNA damage^33^. The link between DNA damage and autophagy in thyroid cancer cells during ferroptosis has not yet been explored, and future mechanistic studies are needed to understand the exact relationship between the autophagy and DNA damage.

Previous studies have demonstrated that rapamycin, an inhibitor of mTOR, reduces GPX4 protein expression and promotes ferroptosis and autophagy^9,10^. In this study, we showed that RSL3 induces autophagy through the suppression of mTOR signaling. The p70S6 kinase is a protein, when phosphorylated, is known to induce protein synthesis at the ribosome and has been used as a marker of activation by mTOR, leading to pro-survival signaling activation^34^. Furthermore, we demonstrated that RSL3 treatment impairs the phosphorylation of other downstream mTOR-related proteins, such as S6 and 4E-BP-1. The increase in LC3A/B protein levels suggests that RSL3 treatment leads PTC cells to autophagy. The restoration of mTOR signaling with Fer1 rescue treatment in the context of RSL3 effects, and the genetic knockdown of GPX4, reaffirm our in vitro findings that RSL3-mediated induction of ferroptosis, mTOR pathway suppression, DNA damage and failure of DNA repair, and autophagy are GPX4-dependent processes in thyroid cancer cells.

In conclusion, GPX4 is highly expressed in PTC and is associated with reduced overall survival. We have shown that effective pharmacologic inhibition of GPX4 in PTC cells with direct inhibitor RSL3 induces a robust activation of ferroptosis, increased DNA damage, impaired DNA damage repair response due to reduced p-NPM1 levels, decreased cell viability and cell migration, as well as suppressed spheroid formation and viability in vitro*.* We identified multi-faceted effects of GPX4 inhibitor, RSL3, in suppressing thyroid cancer cell survival. Previously unreported effects of RSL3, such as degradation of mTOR signaling pathway protein, p70S6K, and reduction of p-NPM1 levels were identified in the present study and warrant further investigation. These initial findings collectively help establish a pre-clinical framework for the future studies involving ferroptosis as a potential therapeutic target in therapy-resistant advanced thyroid cancers.

## Methods

### Computational analysis of human thyroid cancers in the cancer genome atlas database (TCGA)

We queried the TCGA database of PTCs utilizing the Firehouse web browser from the Broad Institute (https://gdac.broadinstitute.org/) and utilized TIMER2.0 (https://cistrome.shinyapps.io/timer/), a publicly available computational software, for analyses of tumor clinical and genomic features^35^. The mRNA expression data was available from 501 thyroid cancer samples and 59 healthy thyroid controls. Both, mRNA expression and clinical outcomes data were available for 488 patients. Overall survival (OS) and disease-free survival (DFS) were analyzed and stratified based on GPX4-expression status of PTC tumors. Data are represented in Log^2^ scale.

### Human thyroid cancer cell lines

Human PTC cell lines K1 and TPC-1 (Sigma-Aldrich, St. Louis, MO), MDA-T32 and MDA-T68 (ATCC, Manassas, VA), and control human thyroid fibroblasts [(HThF); ScienCell Research Laboratories, Carlsbad, CA] were cultured in their respective vendor suggested media.

### Reverse transcription-quantitative polymerase chain reaction (RT-qPCR)

Total RNA was isolated from K1, MDA-T32, MDA-T68, and HThF cells using RNEasy Plus kit (Qiagen, MD). The RT-qPCR analysis was performed on 100 ng of total RNA using iTaq Universal SYBR Green One-Step Kit (Bio-Rad, CA). RT-qPCR was performed on CFX-96 (BioRad, CA). mRNA levels were normalized to *β-Actin*. All primers are listed in Supplementary Table 1.

### Western blot assay

Cells (1 × 10^6^) were grown to 70–80% confluency and treated with DMSO control, (1S,3R)-RSL3 (RSL3, Catalog # 19,288, Cayman Chemical, MI) or Fer1 (Medchemexpress, NJ) as per the experiments. Cells were washed with cold PBS and total proteins were extracted with RIPA buffer containing Halt inhibitors (Thermofisher, MA). A 20–30 μg of total protein were resolved on 4–12% NuPAGE Bis–Tris gels (Thermofisher) and transferred to nitrocellulose membrane using a semi-dry iBlot2 kit (Thermofisher). Blots were blocked with 5% milk in PBS-T (0.01% Tween-20 in phosphate buffered saline). Blots were incubated overnight at 4 °C with primary antibodies and 2 h with HRP-conjugated secondary antibodies at room temperature. Blots were developed using ECL kit (Thermofisher) or scanned using the OdysseyIR scanner (Li-CORBiosciences, NE). All antibodies utilized for Western blotting are listed in Supplementary Table 1. Western blot density quantification was performed using ImageJ software^36^ and are displayed in Supplementary Fig. 1. Some of these Western blots presented in the study were processed using either the mouse or rabbit IRdye conjugated secondary antibodies and scanned with OdysseyIR scanner (Li-CORBiosciences, NE).

### Immunofluorescent staining

The K1 thyroid cancer cells (3 × 10^4^) were plated in 2-chambered slides and treated with DMSO, RSL3 (0.1 μM), or Fer1 (5 μM) for 6 h. Cells were washed and fixed with methanol (100%) and permeabilized with Triton X-100 (0.5%) in PBS. Cells were blocked with 5% BSA in PBS and incubated with primary antibodies overnight. If a second primary antibody was used, the cells were incubated either for 2 h at room temperature or overnight at 4ºC. Cells were washed and incubated with Cy-3 or Cy-5 conjugated secondary antibodies (Jackson ImmunoResearch Laboratories, West Grove, PA and Thermofisher). Fluorescent images were captured with the Zeiss LSM 510 Meta Inverted Confocal Microscope through the Vanderbilt University Cell Imaging Shared Resource. Immunofluorescent images were processed with ImageJ processing software and quantification was performed using the ImageJ cell counter feature^36^. Quantification of TfR1 expression on K1 cells was performed by determining the percentage of TfR1 + cells in each of three low magnification fields (20x). The γ-H2AX expression was determined by quantifying the number of foci in 50 K1 cells under each condition.

### Lipid peroxidation assay

Lipid peroxidation staining assay was performed using BODIPY 581/591 C11 dye (Thermofisher, MA) as previously described^19^. The K1 thyroid cancer cells (1.5 × 10^5^) were treated with RSL3 (3 μM), Fer1 (5 μM) or DMSO control for 3.5 h. After incubation with the dye for 30 min, microscopy images were obtained using the Keyence Fluorescent Microscope BZ-X800 (Keyence, IL).

### Reactive oxygen species (ROS) assay

Cells (1 × 10^4^) were plated in a 96-well white-edged plate and treated with RSL3 (0.1–2.0 μM) for 18 h. H_2_0_2_ substrate was added and incubated for 6 h. ROS assay was performed using ROS-Glo H_2_0_2_ assay kit (Promega, MI) according to the manufacturer’s instructions. Luminescence was measured on GloMax96 microplate reader (Promega, WI).

### Tumor cell viability assay

Cells (2.5 × 10^4^) were plated in a 96-well white edged plate and treated with various concentrations of RSL3 (0.005–2.0 μM) or DMSO for 48 h. Viability assay was performed using CellTiter-Glo luminescent cell viability assay kit (Promega, WI). Luminescence was measured on GloMax96 microplate reader (Promega, WI). Viability assay was also performed with combination of RSL3 and Fer1 (5 μM) or ZVAD (50 μM).

### Tumor spheroid formation assay

K1 thyroid cancer cells (1 × 10^4^) were seeded into a 96-well ultra-low-binding plate in organoid-specific media. After 24 h, cells were treated with DMSO control or RSL3 (0.1–1.0 μM) and incubated for 7 days to allow for tumor spheroid formation. After tumor spheroids were visible in culture, brightfield microscopy images (× 20 magnification) were obtained using Cytation 5 Cell Imaging Multi-Mode Reader (BioTek, VT).

### Tumor spheroid viability assay

K1 cells were incubated to form spheroids (~ 7 days) in ultra-low binding 96-well plates for viability assay and in ultra-low binding 24-well plate for imaging with PI staining. Spheroids were treated with RSL3 and Fer1 for 48 h. Viability of spheroids were determined using CellTiter-Glo 3D cell viability assay kit (Promega). To visualize cell death in the spheroids. 20 μg of propidium iodide (PI) was added for 30 min and microscopic images were taken using Keyence Fluorescent Microscope BZ-X800.

### Migration scratch assay

Thyroid cancer cells (2 × 10^5^) were cultured in 24-well plate to 80–90% confluency. A scratch was made with sterile pipet tip and detached cells were removed by washing with PBS. Microscopic images of scratch were taken and considered as time point zero. Cells were treated either with DMSO or various concentrations of RSL3 (0.5–3.0 μM) for 48 h. Representative images were taken at 48 h.

### GPX4 genetic silencing in vitro

The lentiviral plasmids, either non-targeted vector (pLKO.1-shNT) or pLKO.1-shGPX4 plasmid DNAs (Sigma-Aldrich) were transfected along with packaging and envelop plasmids psPAX2 and pMD2.G into HEK293T cells. The non-targeted or pLKO.1-shGPX4 lentiviral particles were transiently transduced into K1 cells. After 72 h, cells were harvested, washed, and lysed in lysis buffer and Western blot analysis was performed. GPX4 knockdown levels were confirmed using a GPX4 antibody. All methods involving human material were carried out in accordance with the Declaration of Helsinki guidelines and regulations.

### Statistical analysis

Computational survival analysis of OS and DFS based on *GPX4* expression was completed using a log-rank test of the survival curves generated using the Kaplan–Meier method. Analyses of TfR1 expression and γ-H2AX nuclear foci quantification were performed using nonparametric Mann–Whitney test*.* Statistical analyses were performed using GraphPad Prism 9 Software (San Diego, CA). Throughout the manuscript, statistical significance is designated as: ns(*p* ≥ 0.05), *(* p* < 0.05), **(*p* < 0.01), and ***(*p* < 0.001).

## Supplementary Information

Supplementary Information 1.

Supplementary Information 2.

## Data Availability

The data that support the findings of this study are openly available in TCGA database of PTCs utilizing the Firehouse web browser from the Broad Institute (https://gdac.broadinstitute.org/) using TIMER2.0 (https://cistrome.shinyapps.io/timer/), a computational software for analyses of tumor immunologic, clinical, and genomic features^35^. The data that support the findings of this study are available from the corresponding author, NB, upon reasonable request.
