# Supplementary material for: Glutathione peroxidase 4 inhibition induces ferroptosis and mTOR pathway suppression in thyroid cancer
Source: Sci Rep. 2022 Nov 12;12:19396. doi: 10.1038/s41598-022-23906-2 (PMC9653479; doi:10.1038/s41598-022-23906-2)

Fig1E Unprocessed Western Blot Images

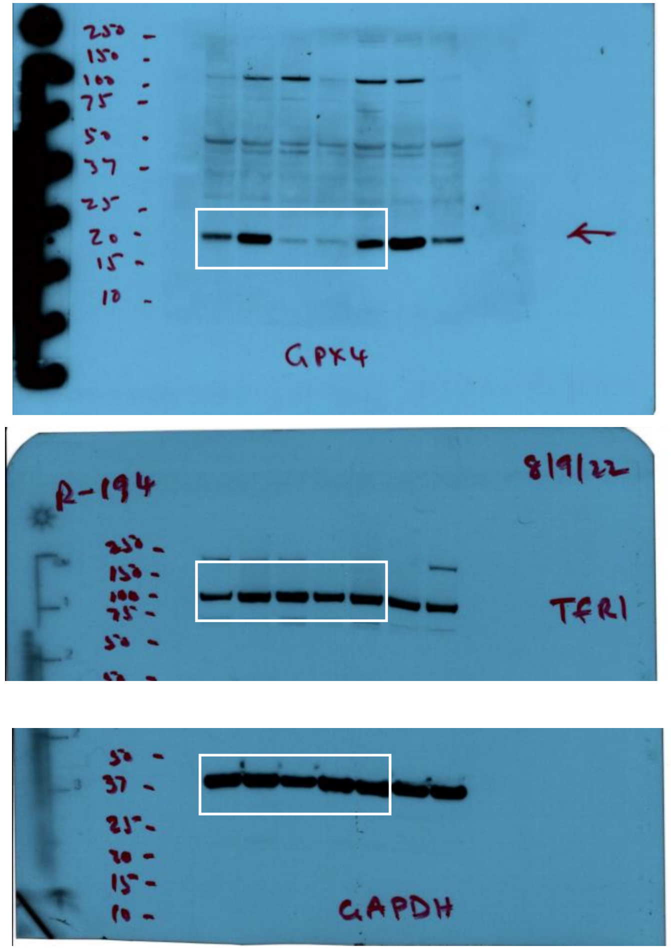

Fig2B Unprocessed Western Blot Images

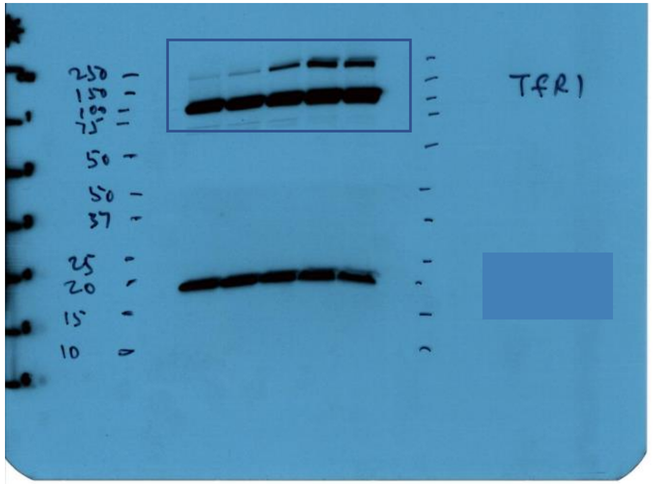

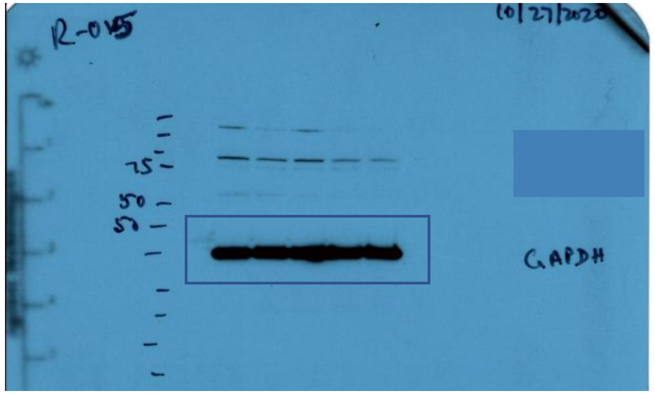

Fig4C Unprocessed Western Blot Images

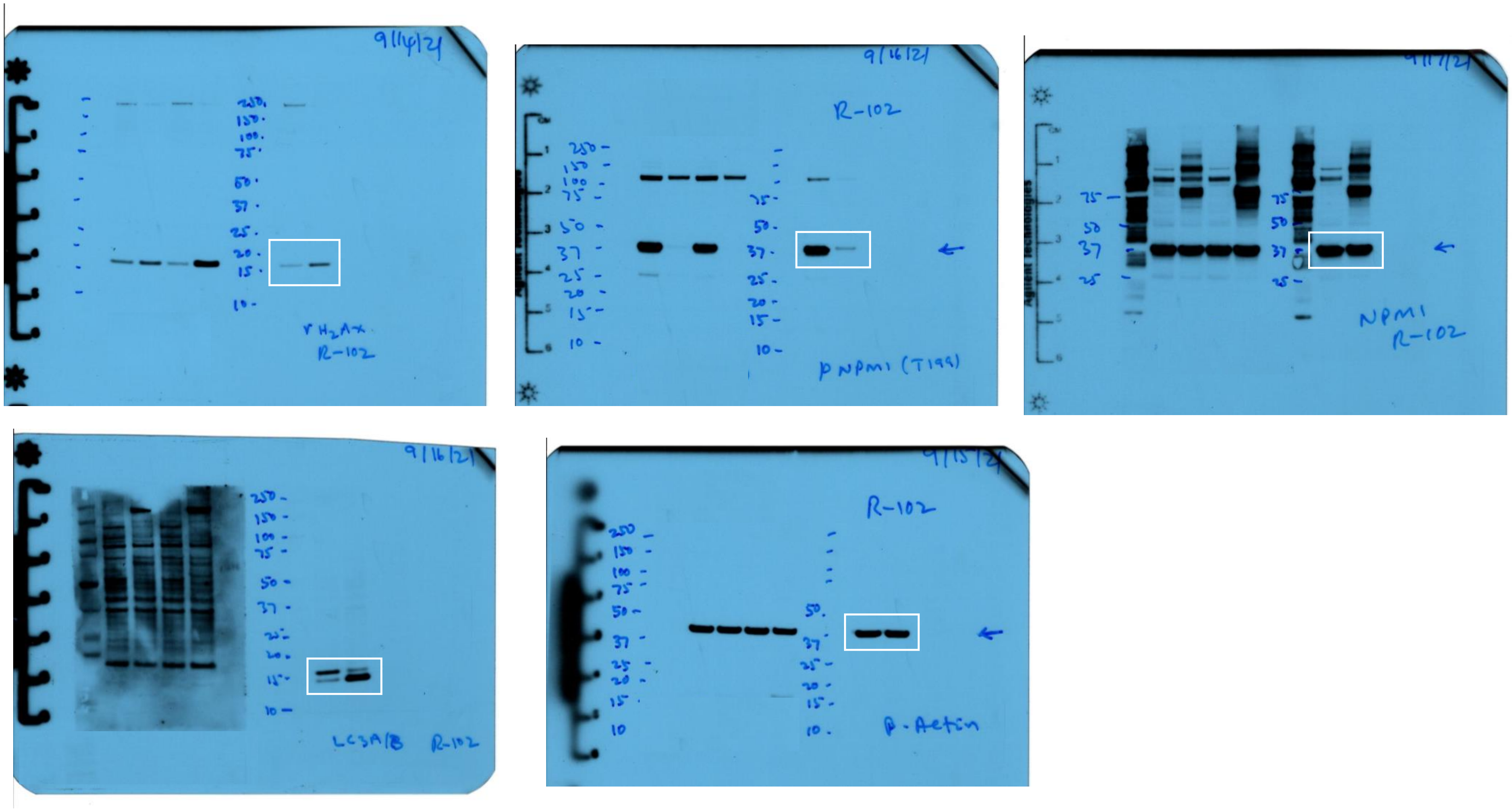

Fig 4F Unprocessed Western Blot Images

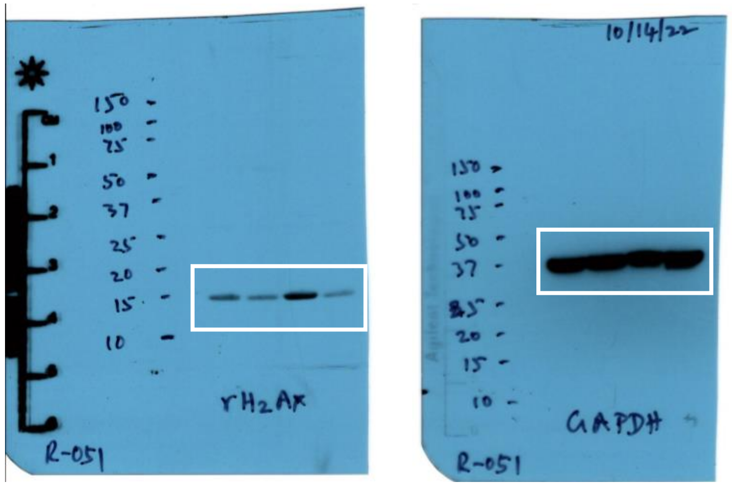

Fig5A Unprocessed Western Blot Images

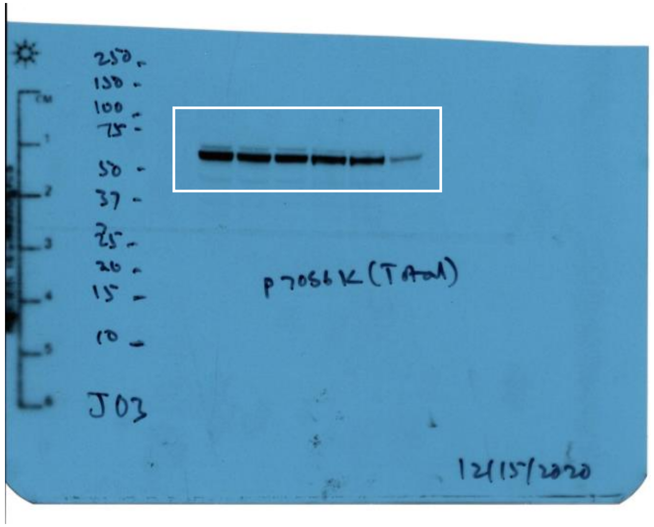

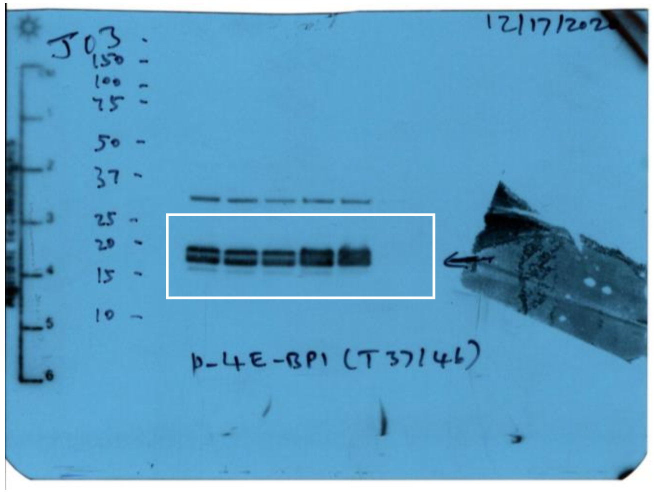

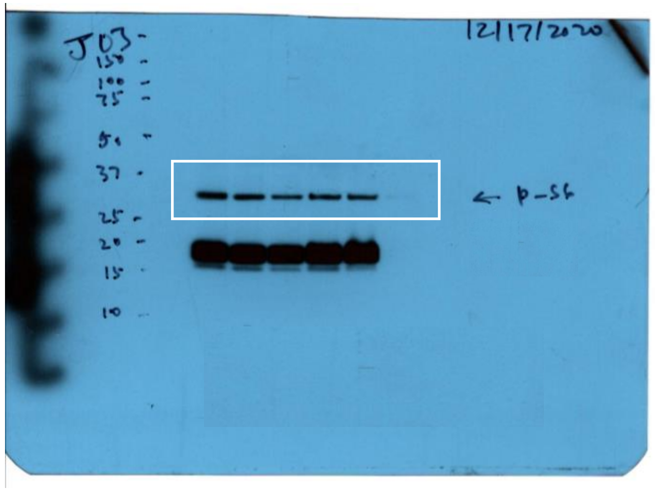

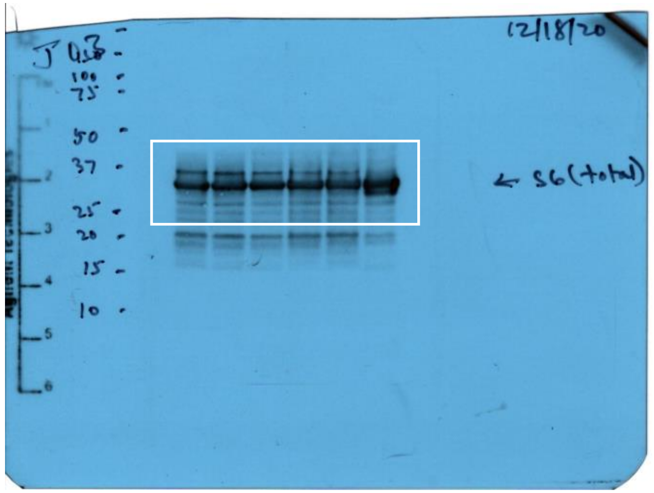

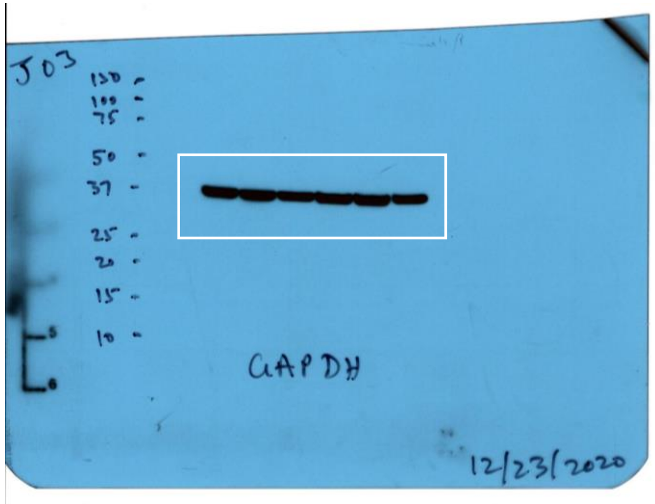

Fig5B Unprocessed Western Blot Images

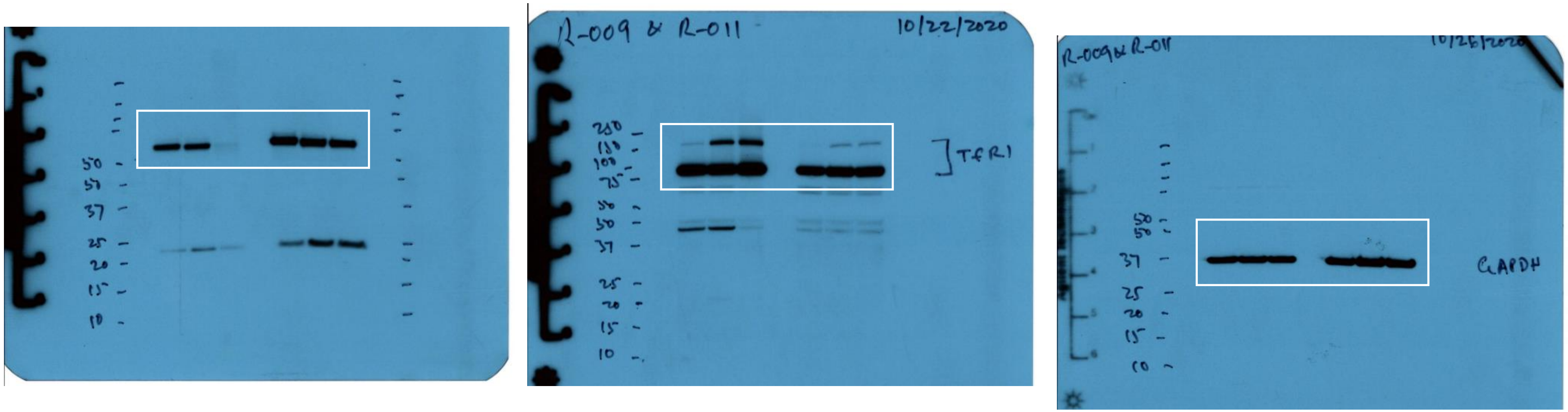

Fig 5C Unprocessed Western blots

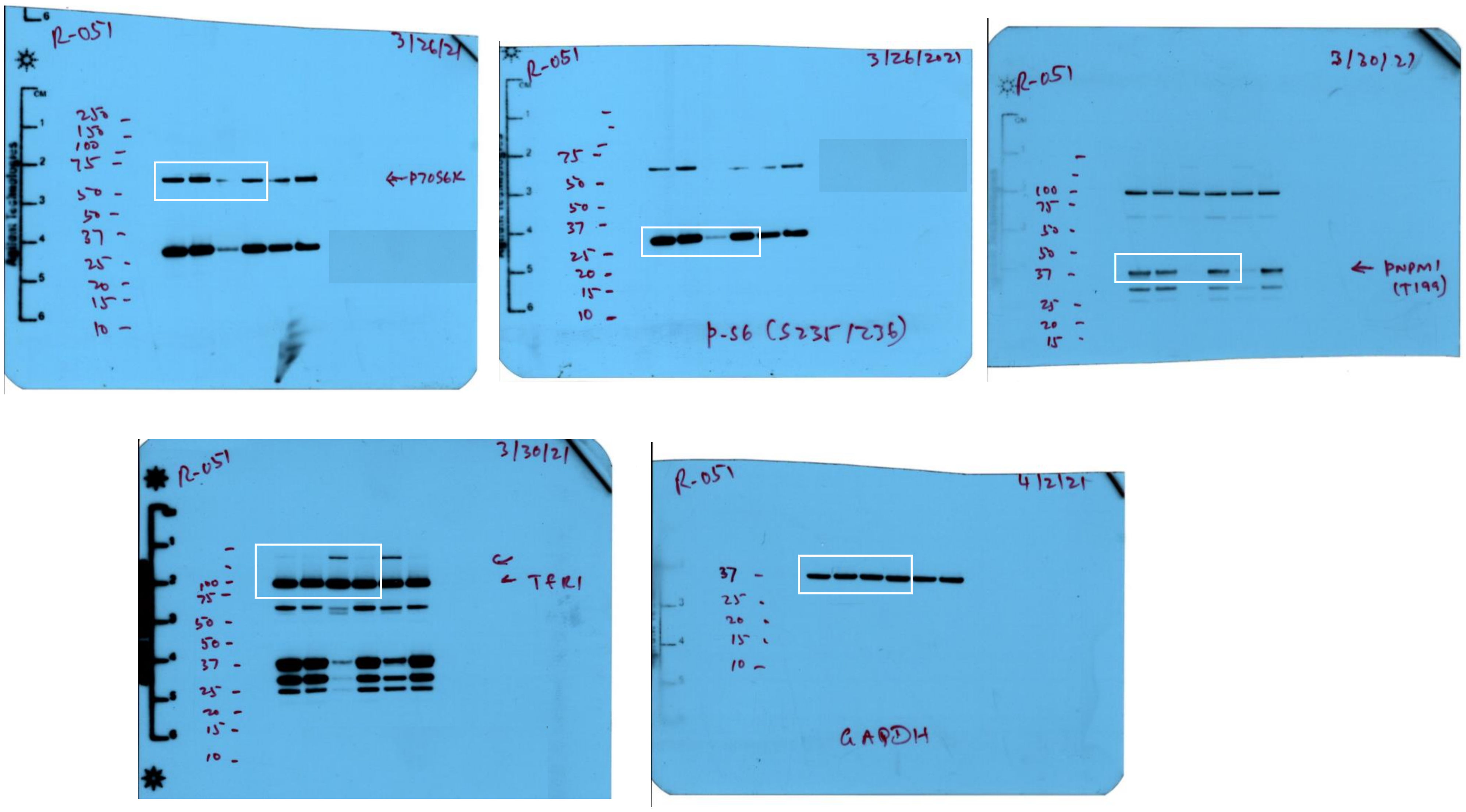

Fig5D Unprocessed Western Blot Images

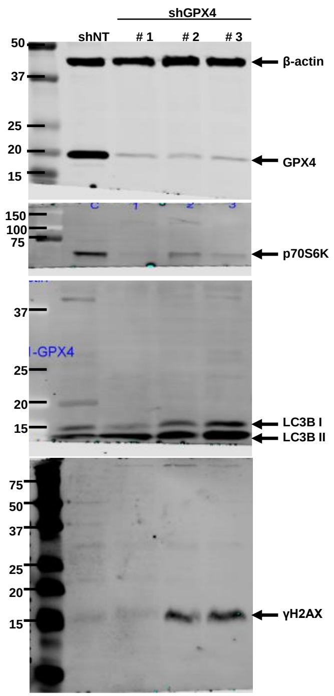

Note: These are original digital scans of WB using LiCor system.

Supplemental Figure 2 Unprocessed Western Blot Images

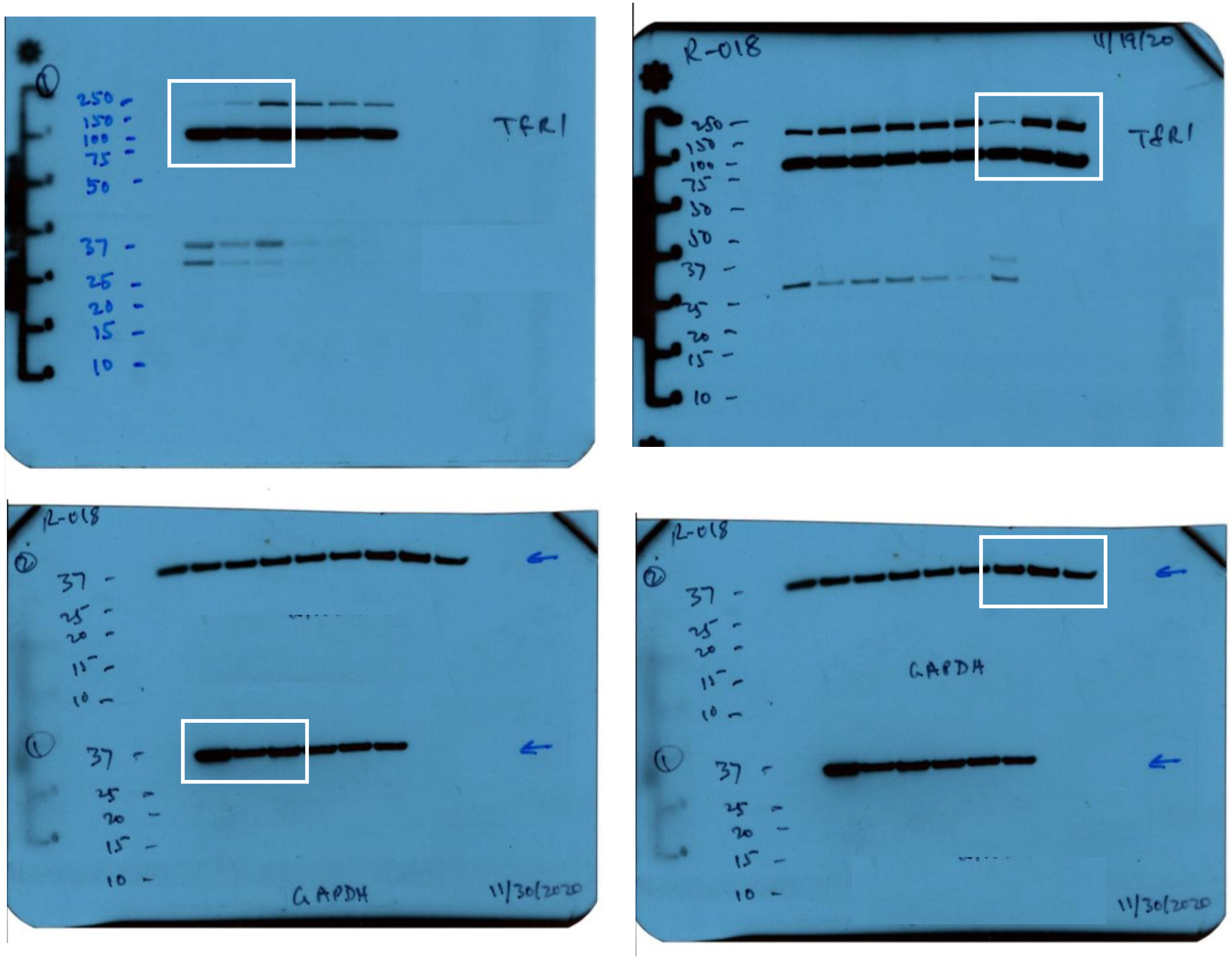

Supplemental Figure 5 Unprocessed Western Blot Images

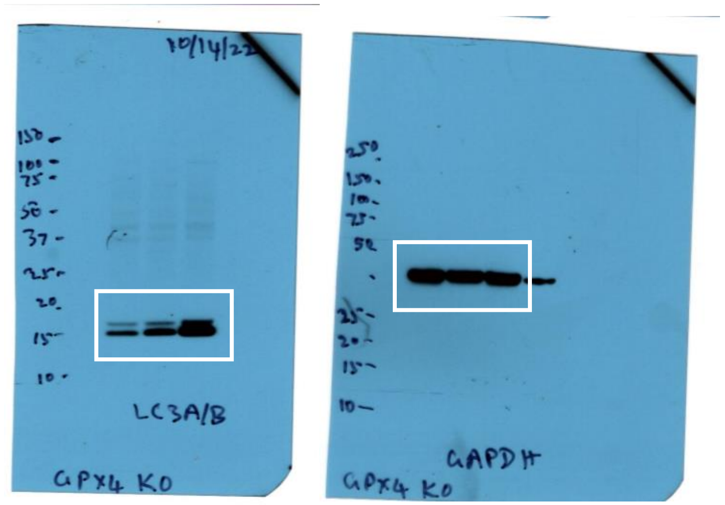

Supplement: Supplementary file 1 — Supplementary Information 1. [file 41598_2022_23906_MOESM1_ESM.pdf]
