# Supplementary material for: Glutathione peroxidase 4 inhibition induces ferroptosis and mTOR pathway suppression in thyroid cancer
Source: Sci Rep. 2022 Nov 12;12:19396. doi: 10.1038/s41598-022-23906-2 (PMC9653479; doi:10.1038/s41598-022-23906-2)
Supplement: Supplementary file 2 — Supplementary Information 2. [file 41598_2022_23906_MOESM2_ESM.pdf]

SUPPLEMENTAL FIGURES

Supplemental Figure 1. ***Western Blot Densitometry Analyses.*** **A.** Densitometry of Figure 1E. Bar graph quantifies increased protein levels of GPX4 in K1 cells compared to other thyroid cancer cell lines, and increased TfR1 expression in all thyroid cancer cell lines compared to HThF. **B.** Densitometry of Figure 5C. Bar graph quantifies RSL3-mediated (3 µM) effect on mTOR signaling pathway proteins (p70S6K, pS6), DNA damage repair response (pNPM1), and induction of ferroptosis (TfR1) in K1 cells. Ferrostatin-1 (Fer1) (5 µM) treatment rescues expression of these proteins in thyroid cancer cells.

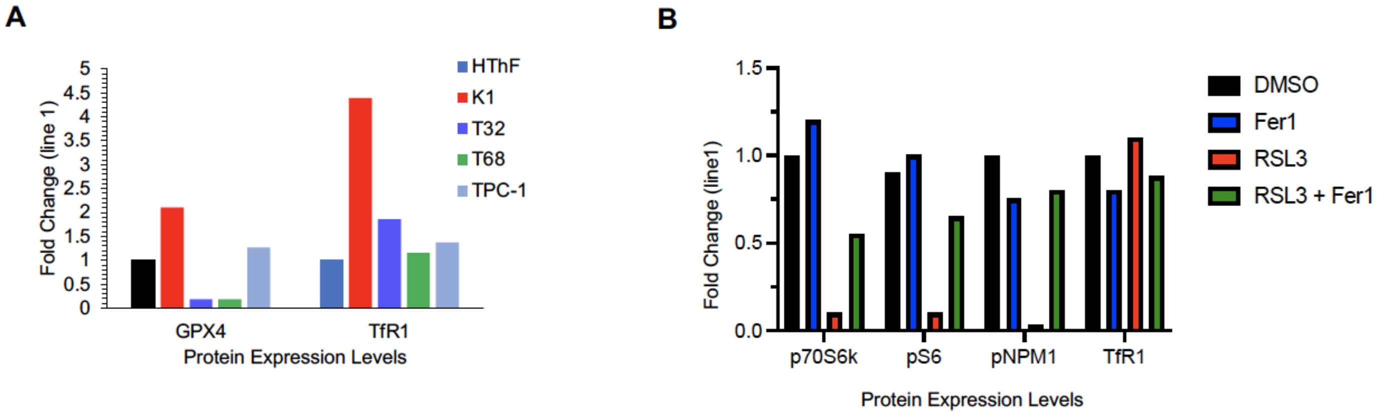

Supplemental Figure 2. ***RSL3 Induces TfR1 Protein Expression in Dose-dependent Manner in Thyroid Cancer Cells.*** **(A)** MDA-T32 and (**B**) MDA-T68 thyroid cancer cells.

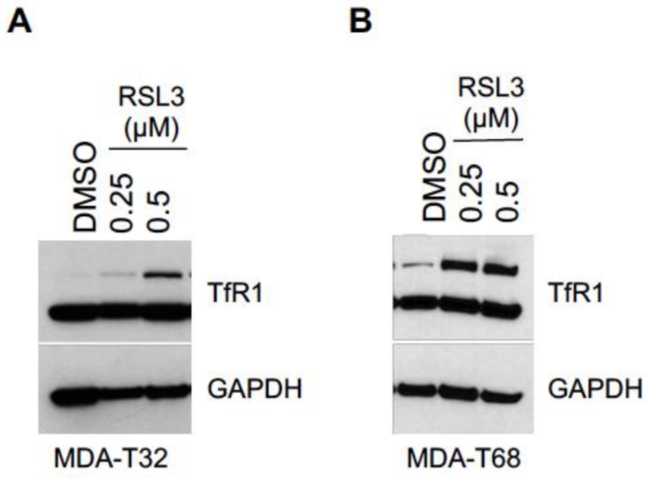

Supplemental Figure 3. *Reduced p70S6K Expression in RSL3-treated K1 Cells.* Immunofluorescent staining demonstrates p70S6K reduction with RSL3 treatment when compared to DMSO controls (p70S6K=green, DAPI=blue, x40 magnification). Images were taken using Keyence Fluorescent Microscope BZ-X800 (Keyence, IL).

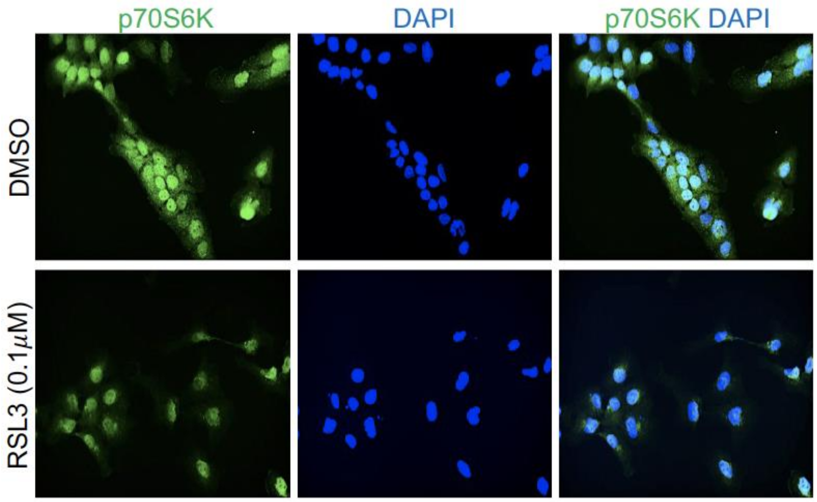

Supplemental Figure 4. *Ferrostatin-1 Rescues K1 Cells from RSL3-induced Morphological Changes In Vitro.* K1 cells were treated with RSL3 alone or in combination with Ferrostatin-1 or ZVAD and brightfield images were captured (x20 magnification).

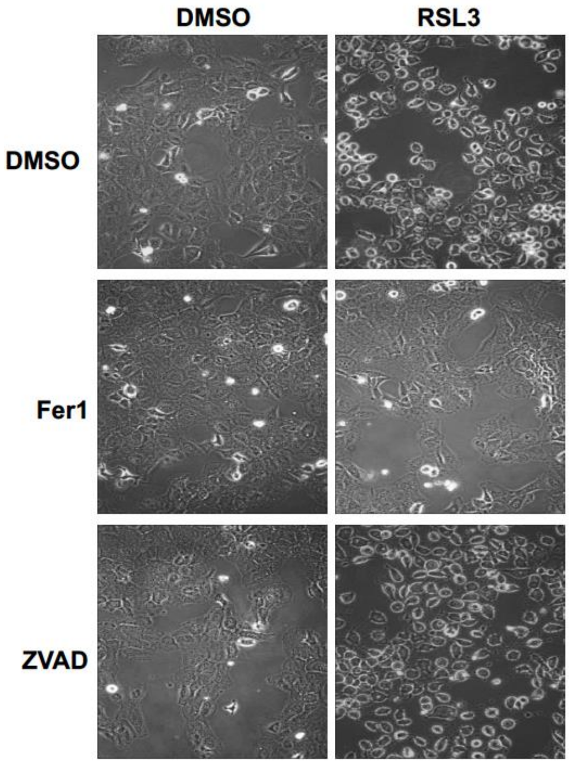

Supplemental Figure 5. ***Increased Levels of LC3A/B in GPX4 knockout K1 cells.***

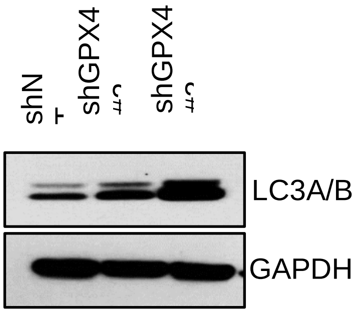

Supplemental Table 1. List of Primers, Antibodies, Chemicals, and shRNA Clones.

qPCR Primers

Target Gene	Forward (5’ to 3’)	Reverse (5’ to 3’)
TfR1	CTGCTTTCCCTTTCCTTGCATATT	GCTCGTGCCACTTTGTTCAACT
GPX4	TTCCCGTGTAACCAGTTCGG	GGTGAAGTTCCACTTGATGGC
β-Actin	GACAGGATGCAGAAGGAGATC	TGCTGATCCACATCTGCTG

Antibodies

Target Protein	Company	Catalog Number	Dilution
GPX4	SantaCruz	sc-166570	1:1000 (WB)
TfR1	ThermoFisher	136800	1:2000 (WB), 1:200 (IF)
p70S6K	Cell Signaling	2709	1:2000 (WB), 1:500 (IF)
pS6	Cell Signaling	4858	1:2000 (WB)
S6	Cell Signaling	2217	1:2000 (WB)
γH2Ax	Sigma/Millipore	05-636	1:1000 (WB), 1:200 (IF)
p-NPM1	Abcam	ab81551	1:2000 (WB), 1:500 (IF)
NPM1	ThermoFisher	325200	1:2000 (WB)
p-4E-BP1	Cell Signaling	2855	1:1000 (WB)
GAPDH	SantaCruz	sc-47724	1:2000 (WB)
LC3A/B	Cell Signaling	12741	1:2000 (WB)
LC3B	Cell Signaling	2775	1:1000 (WB)
β-Actin	SantaCruz	sc-8432	1:2000 (WB)

shGPX4 Clones

Name	Clone	Target Sequence	Reference Sequence
shGPX4 #1	TRCN0000046249	GTGAGGCAAGACCGAAGTAAA	NM_002085
shGPX4 #2	TRCN0000046251	GTGGATGAAGATCCAACCCAA	NM_002085
shGPX4 #3	TRCN0000046252	GCACATGGTTAACCTGGACAA	NM_002085

Chemical Reagents

Name	Company	Catalog Number	Solvent
RSL3	Cayman Chem	19288	DMSO
Ferrostatin 1	Medchemexpress	HY-100579	DMSO
Z-VAD-FMK	AdooQ BioSci	A12373	DMSO
BODIPY™ 581/591 C11	ThermoFisher Sci	D3861	DMSO
